# Familial associations for rheumatoid autoimmune diseases

**DOI:** 10.1093/rap/rkaa048

**Published:** 2020-09-22

**Authors:** Hauke Thomsen, Xinjun Li, Kristina Sundquist, Jan Sundquist, Asta Försti, Kari Hemminki

**Affiliations:** r1 Division of Molecular Genetic Epidemiology, German Cancer Research Center (DKFZ), Heidelberg, Germany; r2 Center for Primary Health Care Research, Lund University, Malmö, Sweden; r3 Bioinformatics and Biostatistics Working Section, GeneWerk GmbH, Heidelberg, Germany; r4 Stanford Prevention Research Center, Stanford University School of Medicine, Stanford, CA, USA; r5 Hopp Children's Cancer Center (KiTZ), German Cancer Research Center (DKFZ); r6 Division of Pediatric Neurooncology, German Cancer Consortium (DKTK); r7 Division of Cancer Epidemiology, German Cancer Research Center (DKFZ), Heidelberg, Germany; r8 Faculty of Medicine and Biomedical Center in Pilsen, Charles University in Prague, Pilsen, Czech Republic

**Keywords:** rheumatoid arthritis, ankylosing spondylitis, Sjögren’s syndrome, lupus, discordant risks, polyautoimmunity

## Abstract

**Objective:**

Previous studies have shown a familial component in RA and in some other rheumatic autoimmune diseases (RAIDs), but because of the different study designs the risk estimates for familial risks differ extensively. The objective of this study is to identify familial components for RAIDs.

**Methods:**

We collected data on patients diagnosed in Swedish hospitals with RA, AS, PM/DM, SS, SLE and SSc (and scleroderma) and calculated familial standardized incidence ratios (SIRs) for each of these (concordant) and between them (discordant).

**Results:**

The combined number of RAID patients in the offspring population (for whom SIRs were calculated) was 71 544, and in the whole population the number was 152 714, accounting for 19.8% of all autoimmune diseases in Sweden. AS showed the highest concordant familial risk of 18.42, followed by SLE (14.04), SS (8.63), SSc (4.50), PM/DM (4.03) and RA (3.03). There was no sex difference in SIRs. Risks for AS and SLE were 80.28 and 19.53 for persons whose parents and siblings were affected. Discordant risks were far lower than concordant risks, but they were significant for RA with all the other five RAIDs, for SLE and SSc with four RAIDs, for AS and SS with three RAIDs and for PM/DM with two RAIDs, attesting to extensive polyautoimmunity between RAIDs.

**Conclusion:**

The derived familial risks in this nationwide family study on medically diagnosed RAID are compatible with emerging evidence on the polygenic background of these complex diseases. Novel genetic pathways offer new therapeutic targets that alleviate disease onset optimally in high-risk familial patients and others.

**Key messages**

- Rheumatoid autoimmune disease patients accounted for 13.8% of all autoimmune diseases in the offspring population.
- Concordant familial risks for rheumatoid autoimmune diseases were always clearly higher than the discordant risks.
- The result showed extensive familial polyautoimmunity between the rheumatoid autoimmune diseases.

## Introduction

Rheumatic autoimmune diseases (RAIDs) include conditions such as AS, PM/DM, RA, SS, SLE and SSc (and scleroderma). In industrialized countries, their prevalence ranges from the most common one, RA, at 1% to the rare ones, PM/DM and SS, at ∼0.02% [1–4]. Most of them, and particularly SLE and SS, are more common in women compared with men, but SSc does not have a large sex difference and AS is more common in men. In autoimmune diseases (AIDs), dysregulated lymphocytes react against self-antigens by producing autoantibodies and suppressing the normal immune function [5]. In RAIDs the misdirected inflammation affects connective tissue, with a preference for the spine in AS, skin and muscle in DM/PM, joints in RA, joints and internal organs in SLE, salivary and lacrimal glands in SS and skin and internal organs in SSc [6–8]. Diagnosis is based on clinical assessment, supported by investigations, including the finding of autoantibodies in RAIDs (except for AS lacking autoantibodies). Treatments include a wide and expanding range of pharmacological modalities, including anti-inflammatory, cytotoxic, immunomodulating and immunosuppressive agents and B-cell-targeted therapies [9–11]. Family and twin studies have shown that genetic risk factors contribute to the aetiology of RA and some other RAIDs [1, 2, 5, 12–17].

Familial AIDs have been extensively studied using a number of different designs, with vastly differing results. A review published in 2013 surveyed the literature on five common AIDs, including RA and SLE, and summarized the results of 44 studies. The review concluded: ‘Thus, further studies of familial autoimmunity will help in increasing the knowledge about the common mechanisms of autoimmunity’ [13]. Following such recommendations, we used the Swedish medical records on the six RAIDs and calculate familial risks for each of these (concordant) and between them (discordant). Data are presented as proband- and sex-specific familial risks.

## Methods

RAID patients were identified from the Swedish Hospital Discharge Register (years 1964–2012, full national coverage from 1986 onwards) and the Outpatient Register (2001–2012) with any diagnostic codes for RAIDs. Only the first AID diagnosis was included. Of a total of 769 991 patients, 51% were identified from the Inpatient Register and 49% from the Outpatient Register. If a patient was treated only in primary care, they were excluded from the analysis. However, in view of diagnostic verification and treatment planning, such cases were probably very few (see the first paragraph of the Discussion). Various revisions of the International Classification of Diseases (ICD) codes were used for AIDs, as described elsewhere [18]. Family relationships were obtained from the Multigeneration Register, containing the Swedish population in families and spanning more than a century [19]. As family members, only first-degree relatives of offspring–parent pairs and siblings in the offspring generation were considered; the offspring generation was born after 1931, and the parental generation was born any time earlier. By 2012, the offspring generation reached the age of 80 years; siblings can be defined only in the offspring generation. For the parental generation, there was no age limit. For the family history, a register-based definition was used without consideration of the timing of the diagnoses among family members, because it was shown to be preferable in terms of case numbers [20]. Information from the registers was linked at the individual level via the national 10-digit civic registration number. In the linked dataset, civic registration numbers were replaced with serial numbers to ensure the anonymity of all individuals. The study was approved by the Ethical Committee of Lund University.

Standardized incidence ratios (SIRs) were calculated for the offspring generation as the ratio of the observed to expected number of cases. The expected numbers were calculated for all individuals without a first-degree family history of a specific AID (i.e. essentially for the whole Swedish population), and the rates were standardized by 5-year-age, sex, period (groups of 5 years), socioeconomic status and residential area. The follow-up was started in 1964 and continued until 2012. The 95% CI of the SIR was calculated assuming a Poisson distribution. Separate SIRs were calculated for offspring when only a parent, only a sibling, or a parent and a sibling were probands, (i.e. they were diagnosed with concordant RAID). In analysis of discordant RAIDs, bidirectional (i.e. RA–AS and AS–RA) associations were considered.

## Results

The number of RAID patients in the offspring generation (for whom risks were calculated) was 46 256, with a mean diagnostic age (i.e. first hospital contact) of 48.2 years; considering their parents also, the total number was 112 958 (Table 1). PM/DM presented the smallest number of patients: 1384 in the offspring generation and 2668 including the parental generation. Among the offspring patients, AS and SLE patients were the youngest (28.5 and 39.4 years, respectively) and SS patents the oldest (53.5 years). The total AID population amounted to 519 180 patients in the offspring generation of 8.5 million. Thus, RA accounted for 8.9% of all AIDs and was diagnosed in 0.54% of the offspring population. Jointly, RAID patients numbered 71 544 in the offspring population and 152 714 in the whole population, accounting for 13.8 and 19.8% of all AIDs, respectively.

**Table 1:** Number of cases of autoimmune diseases in offspring (*n* = 8 517 416) and in the total population, 1964–2012

	Events in the study population			Number of events in the total population	Percentage of males
	Number	Percentage	Mean age, (s.d.)		
Total	519 180		38.8 (19.5)	769 991	40.2
Subtype					
AS	11 226	2.2	39.6 (13.7)	15 097	65.6
PM/DM	1384	0.3	44.6 (20.9)	2668	46.3
RA	46 256	8.9	48.2 (17.0)	112 958	29.5
SS	5754	1.1	53.5 ( 13.1)	8971	9.3
SLE	5201	1.0	39.4 (15.8)	9566	16.1
SSc	1723	0.3	47.3 (16.5)	3454	21.4

### Concordant familial risks

Familial risks for the RAIDs are shown in Table 2 for offspring whose first-degree relatives (parents or siblings as probands) were diagnosed with concordant RAID. The SIRs differed widely. AS showed the highest risks: 16.12 when parents, 16.57 when siblings, and 80.28 when parents and siblings were probands. Next in rank was SLE, with the respective SIRs of 13.30, 13.55 and 19.53. For RA, the risks were 2.64, 2.89 and 7.17. For PM/DM, only the sibling risk of 7.39 was significant. None of the SIRs between parents–offspring and siblings were significant (i.e. the 95% CIs overlapped).

**Table 2:** Familial risks of concordant autoimmune diseases

Disease	Parent only			Sibling only			Both parent and sibling		
	**Obs.**	**SIR**	**95% CI**	**Obs.**	**SIR**	**95% CI**	**Obs.**	**SIR**	**95% CI**
AS	371	**16.12**	**14.52, 17.84**	509	**16.57**	**15.17, 18.08**	53	**80.28**	**60.12, 105.06**
PM/DM	1	1.35	0.00, 7.72	4	**7.39**	**1.92, 19.11**	0		
RA	3163	**2.64**	**2.55, 2.73**	1945	**2.86**	**2.74, 3.00**	297	**7.17**	**6.38, 8.04**
SS	68	**8.01**	**6.22, 10.16**	107	**9.41**	**7.71, 11.37**	2	**24.69**	**2.33, 90.81**
SLE	118	**13.30**	**11.01, 15.93**	94	**13.55**	**10.95, 16.58**	2	**19.53**	**1.84,71.83**
SSc	5	**4.28**	**1.35, 10.06**	4	**3.92**	**1.02, 10.13**	0		

Bold type indicates that the 95% CI does not include 1.00.

Obs.: observed number of cases; SIR: standardized incidence ratio.

Sex-specific familial risks are shown in Table 3 using any first-degree relatives as probands. The ranking order from Table 2 was led by AS, with a familial risk of 18.42, and followed by SLE (14.04), SS (8.63), SSc (4.50), PM/DM (4.03) and RA (3.03). There was no single-sex difference in SIRs. For RA, the male and female SIRs differed only marginally (2.93 *vs* 3.07). For SS and SLE, with a large female excess of cases, female risk was slightly higher for SS (8.96 *vs* 5.57) but male risk was slightly higher in SLE (15.39 *vs* 13.83).

**Table 3:** Familial risks for concordant rheumatoid autoimmune diseases

	Both sexes					Men					Women				
Autoimmune disease	SIR	Obs.	95% CI		*P*-value	SIR	Obs.	95% CI		*P*-value	SIR	Obs.	95% CI		*P*-value
AS	**18.42**	**868**	**17.21**	**19.66**	**0.00**	**17.47**	**543**	**16.03**	**18.97**	**0.00**	**20.26**	**325**	**18.12**	**22.52**	**0.00**
PM/DM	**4.03**	**5**	**1.27**	**8.35**	**0.00**	**5.77**	**3**	**1.09**	**14.15**	**0.01**	2.78	2	0.26	7.97	0.24
RA	**3.03**	**5418**	**2.95**	**3.11**	**0.00**	**2.94**	**1611**	**2.80**	**3.08**	**0.00**	**3.07**	**3807**	**2.98**	**3.17**	**0.00**
SS	**8.63**	**158**	**7.34**	**10.03**	**0.00**	**5.57**	**10**	**2.65**	**9.55**	**0.00**	**8.96**	**148**	**7.58**	**10.47**	**0.00**
SLE	**14.04**	**204**	**12.18**	**16.03**	**0.00**	**15.30**	**32**	**10.46**	**21.07**	**0.00**	**13.83**	**172**	**11.84**	**15.97**	**0.00**
SSc	**4.50**	**9**	**2.04**	**7.91**	**0.00**	2.22	1	0.00	8.70	0.75	**5.16**	**8**	**2.20**	**9.35**	**0.00**

Obs.: observed number of cases; SIR: standardized incidence ratio.

We also calculated the risk between spouses, but the number of cases was low for RAIDs other than RA. For RA, the SIR was 1.16 (*n* = 593, 95% CI: 1.07, 1.26).

### Discordant familial risks

We analysed familial risks between the five discordant RAIDs in Table 4. RA was associated with all other five RAIDs, with SIRs ranging from 1.35 to 2.08. SLE and SSc were associated with four RAIDs each; SIRs ranged from 1.80 to 3.61 for SLE and from 1.75 to 2.71 for SSc. AS and SS were associated with three RAIDs each; SIRs ranged from 1.58 to 1.69 for AS and from 1.78 to 2.83 for SS. PM/DM was associated with RA (1.35) and SLE (2.28).

**Table 4:** Familial risks for discordant rheumatoid autoimmune diseases

		Both sexes			
Subtypes of AID in offspring	Family history of AID	SIR	Obs.	95% CI	*P*-value
AS	PM/DM	1.45	14	0.79, 2.31	0.17
AS	RA	**1.61**	**638**	**1.48, 1.73**	**0.00**
AS	SS	**1.58**	**57**	**1.19, 2.01**	**0.00**
AS	SLE	1.35	41	0.97, 1.79	0.06
AS	SSc	**1.69**	**21**	**1.05, 2.50**	**0.02**
PM/DM	AS	1.05	6	0.38, 2.05	0.92
PM/DM	RA	**1.35**	**73**	**1.06, 1.68**	**0.01**
PM/DM	SS	0.67	3	0.13, 1.65	0.55
PM/DM	SLE	**2.28**	**9**	**1.03, 4.00**	**0.02**
PM/DM	SSc	1.26	2	0.12, 3.60	0.80
RA	AS	**1.92**	**340**	**1.72, 2.13**	**0.00**
RA	PM/DM	**1.47**	**60**	**1.12, 1.87**	**0.00**
RA	SSS	**1.69**	**243**	**1.49, 1.91**	**0.00**
RA	SLE	**2.08**	**264**	**1.83, 2.33**	**0.00**
RA	SSc	**1.35**	**71**	**1.06, 1.68**	**0.01**
SS	AS	1.38	31	0.94, 1.91	0.07
SS	PM/DM	0.94	5	0.30, 1.94	0.90
SS	RA	**1.78**	**409**	**1.61, 1.95**	**0.00**
SS	SLE	**2.83**	**46**	**2.07, 3.71**	**0.00**
SS	SSc	**2.20**	**15**	**1.23, 3.46**	**0.00**
SLE	AS	1.26	28	0.84, 1.77	0.23
SLE	PM/DM	**2.42**	**11**	**1.20, 4.06**	**0.00**
SLE	RA	**1.80**	**346**	**1.62, 2.00**	**0.00**
SLE	SSS	**3.61**	**61**	**2.76, 4.58**	**0.00**
SLE	SSc	**3.39**	**20**	**2.07, 5.04**	**0.00**
SSc	AS	**2.22**	**15**	**1.24, 3.49**	**0.00**
SSc	PM/DM	1.30	2	0.12, 3.73	0.77
SSc	RA	**1.75**	**117**	**1.45, 2.08**	**0.00**
SSc	SS	**2.60**	**14**	**1.42, 4.14**	**0.00**
SSc	SLE	**2.71**	**13**	**1.44, 4.38**	**0.00**

AID: autoimmune disease; Obs.: observed number of cases; SIR: standardized incidence ratio.

We also analysed sex-specific discordant associations, but given that none of these was significant the results are not shown.

### Summarizing concordant and discordant associations

**Fig. 1:**
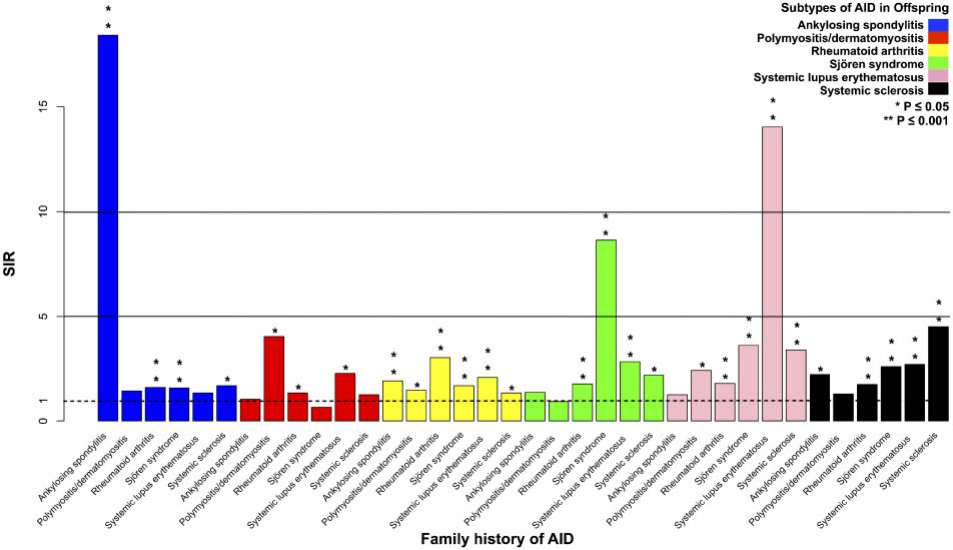
Familial associations of concordant and discordant rheumatic autoimmune diseases *Statistically significant associations. The *P*-values are indicated by asterisks on top of the bars. AID: autoimmune disease; SIR: standardized incidence ratio.

Significant associations for the six RAIDs are shown in Fig. 1. The concordant risk of AS is prominent, compared with its modest discordant risks. This is in contrast to RA and SSc, with smaller differences between concordant and discordant risks. RA was the significant proband partner with the five other RAIDs (i.e. the SIRs were reciprocally increased with RA and other RAIDs). Disregarding associations with RA, AS was associated only with SSc and PM/DM only with SLE; SS, SLE and SSc were all reciprocally associated.

## Discussion

Incidence data for common diseases are liable to biases depending on the source of the data (hospital, hospital discharge, insurance data etc.), diagnostic criteria and level of reporting [21]. Family studies add another level of complexity, because family histories are usually obtained anecdotally by interview; reporting accuracies even for relatively well-defined diseases, such as cancer, show high variability, let alone for diseases, such as AID, where diagnostic criteria (such as ICD codes) have changed over time [22–24]. In the Swedish Multigeneration Register, the national family relationships are unbiased and complete [19]. The advantages of using relatively recent national hospital discharge and outpatient data include high diagnostic accuracy [21]. Given that hospitalizations in Sweden normally require a doctor’s pass from primary care, each patient is seen by at least two medical doctors, of whom the one in the hospital is likely to be a specialist [2]. An ad hoc study on close to 1000 hospitalized RA patients found that some 90% of the patients fulfilled the RA criteria of the ACR [25].

To emphasize the above point about fallacies in family data on AIDs, some examples can be taken from the review of five common AIDs by Cárdenas-Roldán *et al.* [13]. They list three US studies reporting familial risks of concordant RA (the results are given as a relative risk, incidence among first-degree family members compared with population incidence): 2.0, 7.8 and 18.7. Our SIR was 3.03. Two studies reported risks of SLE in RA families: 64 and 28; our SIR was 2.08. Two studies reported risks of RA in SLE families: 390 and 225; our SIR was 1.80. We have no possibility of explaining such high reported familial risks, but high risks would be compatible with a Mendelian genetic background, characterized by high-penetrant genes causing disease in every generation. In contrast, RAIDs are typically non-Mendelian complex diseases on a polygenic background [26]. A polygenic background would imply that the frequencies of risk alleles is widely distributed in the population that has been used to calculate polygenic risk scores based on the number of risk alleles [27]. The high overall risk for AS and SLE might imply a particularly strong polygenic influence, with >100 known genes, and in AS with the HLA allele B27 and its subtypes, and in SLE with non-HLA genes [28, 29].

Polygenic models predict that families with many risk alleles show a high familial risk, which is commensurate with the results in families where both a parent and a sibling were affected (SIR 80.28 in AS and 7.17 in RA). Complex diseases also have environmental risk factors, for which we showed evidence through spouse correlation in RA. The SIR was, however, modest (1.16), and for the other RAIDs spousal case numbers were small for reliable risk estimation. Considering the large sex differences in the incidence of some RAIDs, it was surprising that no single familial risk showed a sex-specific difference.

Polyautoimmunity is a common feature of many AIDs, and particularly among rheumatoid and thyroid AIDs [30, 31]. The present results attest to shared familial risks between RAIDs, because RA was associated with all other RAIDs, and SLE and SSc were associated with four of five discordant RAIDs. The genetic basis of such pleiotropy is understood, in part, through extensively shared low-risk genes [32–36]. A recent genome-wide meta-analysis of autoantibody-positive RAIDs (i.e. all those of the present study except for AS) revealed 26 independent non-HLA significantly associated genetic risk loci [37]. Extensive genetic sharing was evident, in that 85% of the associated variants were shared by at least three diseases. Many of the shared loci were related to immune processes, such as interferon signalling and B- and T-cell-related immune functions, offering possible therapeutic targets [37]. Some studies have pointed out greater sharing of risk loci among the autoantibody-positive or among seronegative diseases than between these two groups [26, 38]. In line with these findings, AS had only three modest discordant associations (ranging from 1.45 to 1.69), although its concordant risk of 18.42 was the highest observed; our study included only AS as an autoantibody-negative disease.

The strengths of the study were the overall large numbers of patients diagnosed in a standard way in a high-level health-care system accessible to the population at large without economic barriers. Limitations include low patients numbers for rare RAIDs and the relatively short follow-up time (2001–2012) of patients from the Outpatient Register. We had no primary care data; however, this guaranteed a defined level of diagnostic accuracy, provided by the specialist wards.

In summary, the present study showed that RAID patients accounted for 13.8% all AIDs in the offspring population and 19.8% in the whole population. RA alone accounted for 8.9% of all AIDs in the offspring population, and it was diagnosed in 0.54% of this population. The results provided conclusive quantitative familial risk estimates for RAIDs and between RAIDs. Concordant familial risks were high for all RAIDs, but particularly for AS and SLE. The discovery of multiple genetic pathways underlying these diseases has pointed out new therapeutic targets that might help with disease intervention in family members at an early stage. The result showed extensive familial polyautoimmunity between these diseases but also specificity, in that the concordant familial risks were always clearly higher than the discordant ones.

*Funding*: The study was supported by the European Union’s Horizon 2020 research and innovation programme, grant no. 856620.

*Disclosure statement*: The authors have declared no conflicts of interest.
